# Field-recorded data on the diet of six species of European *Hydromantes* cave salamanders

**DOI:** 10.1038/sdata.2018.83

**Published:** 2018-05-15

**Authors:** Enrico Lunghi, Fabio Cianferoni, Filippo Ceccolini, Manuela Mulargia, Roberto Cogoni, Benedetta Barzaghi, Lorenzo Cornago, Domenico Avitabile, Michael Veith, Raoul Manenti, Gentile Francesco Ficetola, Claudia Corti

**Affiliations:** 1 Universität Trier Fachbereich VI Raum-und Umweltwissenschaften, Biogeographie, Universitätsring 15, 54286 Trier, Germany; 2 Museo di Storia Naturale dell'Università degli Studi di Firenze, Sezione di Zoologia "La Specola", Via Romana 17, 50125 Firenze, Italia; 3 Natural Oasis, Via di Galceti 141, 59100 Prato, Italia; 4 CNR-IBAF Consiglio Nazionale delle Ricerche, Istituto di Biologia Agroambientale e Forestale, Via Salaria km 29,300, 00015 Monterotondo (Roma), Italia; 5 Via Isalle 4, 08029 Siniscola, Nuoro, Italia; 6 Unione Speleologica Cagliaritana, Via A. Scarlatti, 11, 09045 Quartu Sant'Elena (CA), Italia; 7 Department of Environmental Sciences and Policy, Università degli Studi di Milano, via Celoria 20, 20133 Milano, Italia; 8 Università degli Studi di Bologna, via Selmi 3, 40126 Bologna, Italy; 9 Univ. Grenoble Alpes, CNRS, Laboratoire d’Écologie Alpine (LECA), F-38000 Grenoble, France

**Keywords:** Behavioural ecology, Herpetology

## Abstract

The availability of data on the feeding habits of species of conservation value may be of great importance to develop analyses for both scientific and management purposes. Stomach flushing is a harmless technique that allowed us to collect extensive data on the feeding habits of six *Hydromantes* species. Here, we present two datasets originating from a three-year study performed in multiple seasons (spring and autumn) on 19 different populations of cave salamanders. The first dataset contains data of the stomach content of 1,250 salamanders, where 6,010 items were recognized; the second one reports the size of the intact prey items found in the stomachs. These datasets integrate considerably data already available on the diet of the European plethodontid salamanders, being also of potential use for large scale meta-analyses on amphibian diet.

## Background & Summary

The European cave salamanders, (*Hydromantes*, for further taxonomy information see, (ref. 1)) are the only plethodontid salamanders occurring in Europe^2^. The genus *Hydromantes* includes three species endemic to California and eight species endemic (or sub-endemic) to Italy: five (*H. flavus*, *H. supramontis*, *H. imperialis*, *H. sarrabusensis*, *H. genei*) are endemic to Sardinia, the remaining three (*H. italicus*, *H. ambrosii*, *H. strinatii*) are distributed along Apennine one of the mainland species (*H. strinatii*) is also present in a small part of SW France^2–4^. Moreover, some individuals have been introduced in some European countries^5–7^. Several European *Hydromantes* are all listed as vulnerable or endangered species (I.U.C.N. Red List) and therefore strictly protected by the European laws^8^.

*Hydromantes* species are fully terrestrial salamanders able to exploit several environments, from forest floors to cracks and bare rocks^2,9,10^. However, when local climate becomes unsuitable (too hot and/or harsh), *Hydromantes* salamanders seek refuge underground, where microclimatic conditions are generally suitable all year round^11–14^. In underground environments *Hydromantes* species show stable populations, reaching high densities and being able to carry out most of their biological functions^15–17^. The elusive behavior of these salamanders, combined with the intrinsic complexity of underground environments, often strongly reduces feasibility of data collection; in fact, there is still a paucity of information about biology, ecology and behavior of most of the species of the genus^2^. For example, until recently, the reproduction of *Hydromantes* salamanders was only observed in controlled conditions^2,18,19^; just in the last few years the first observations and researches on *Hydromantes* nesting ecology in natural environments have been performed^16,17^.

One of the most important aspects that need to be studied concerns feeding ecology and diet composition. Until today, studies on the diet of *Hydromantes* were performed only on three European species^20–22^, while for others there is no information^2^. Diet is a dynamic feature characterizing individuals throughout their life^23,24^. Resource requirements depend on life stage and therefore individuals focus their feeding activity on specific food resources^25–27^. Within species range, populations likely occupy areas characterized by different resource assemblages, which in turn can shape species diet at local level^28^. Moreover, seasonality may produce food resource fluctuations, forcing species to adapt their feeding habits to the available ones^29,30^. Finally, individual diet can be affected by the presence of competitors^31–33^. The availability of exhaustive data on the different species diet may be of fundamental importance for ecological and zoological researches in order to define species patterns and strategies.

In the present work, we report a large dataset on the diet of six European *Hydromantes* species (*H. flavus*, *H. supramontis*, *H. imperialis*, *H. sarrabusensis*, *H. genei* and *H. ambrosii*), considering different seasons and numerous populations. For most of these species, as mentioned above, no data on the diet and feeding behavior are available. During a three-year survey, we analysed the stomach contents of different salamanders populations, sampling individuals from underground environments. We produced two different datasets: one contains data on the salamanders’ stomach contents, while the second the maximum length of the intact prey items. Future studies are planned to include data on *H. strinatii* and *H. italicus* in the dataset, and increase the number of sampled populations. The datasets can also be combined with those of other amphibian species, in order to compare local and macro-scale information.

## Methods

### Experimental design

**Figure 1:**
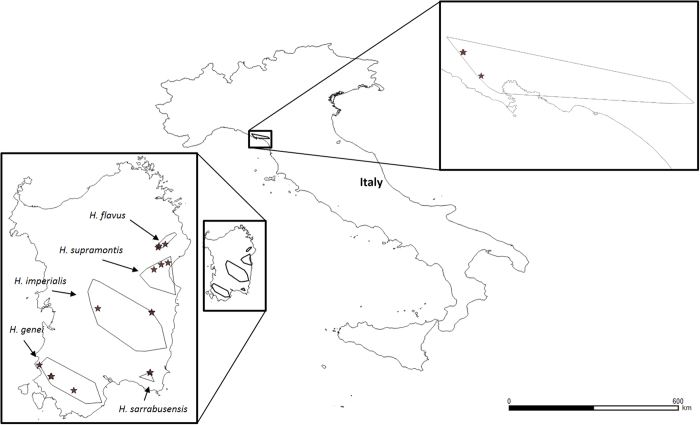
Map of the study area. The distribution of each studied species (polygons obtained combining published and unpublished data^2,40^) and the studied populations (stars). Maps were created with the program QGIS using data from http://www.diva-gis.org/gdata.

We adopted the following methodology to collect the data of the two datasets (Data Citation 1):

1. We sampled at least 3 populations per species, for a total of 19 different underground sites (i.e., caves) (Fig. 1).
2. Multiple sampling was repeated from 2015 to 2017.
3. Sampling was performed in different seasons: May/June 2016 and 2017 (beginning of the hot season; hereafter spring) and early September 2015 and 2016 (end of the hot season; hereafter autumn). In these periods, salamanders intensify their foraging activity in- and outside the caves^10,11^ for both, the upcoming aestivation and to recover after the summer inactivity period^2^.
4. For each population, we performed a minimum of 3 samplings, in different years and seasons.
5. We sampled at least 170 individuals per species.
6. In each site, we sampled males, females and juveniles (see Salamanders sampling).
7. Stomach contents were obtained by stomach flushing and contents preserved in ethanol 75% (see Stomach flushing).
8. Stomach contents were examined at an optic microscope and, when possible, prey items were counted and ascribed to the lower possible taxonomic level (see Stomach contents analysis).
9. Intact prey items, were measured at the maximum length (see Stomach contents analysis).

### Salamander sampling

All surveys were performed during day time (9 a.m. – 6 p.m.). Caves where explored entirely or up to the point reachable without speleological equipment. We divided the cave environments into parts of 3 linear meters each (hereafter, sector) using a laser meter (Anself RZE-70, accuracy 2 mm), starting from the cave entrance to the maximum reachable point (for further explanation see (ref. 13)). We actively searched and captured salamanders using sterile disposable gloves. Each survey ended when salamanders were no longer observed for 15 min. After capture, salamanders were temporarily placed in sterile fauna boxes until stomach flushing was performed (maximum 2 h); when possible, we registered the position (the cave sector) of the captured salamanders. Salamanders’ snout-vent length (SVL) was measured using a transparent plastic ruler where salamanders. Salamanders were sexed by checking the presence/absence of males’ secondary sexual characters (mental gland and pre-maxillary teeth)^2^. Individuals below the size of the smallest male were considered juveniles, as females differ from juveniles only by body-size, without providing any other recognisable feature^2^. The smallest recorded male’ SVL was 40 mm for *H. ambrosii* and *H. genei*, while 45 mm for all other species.

### Stomach flushing

**Figure 2:**
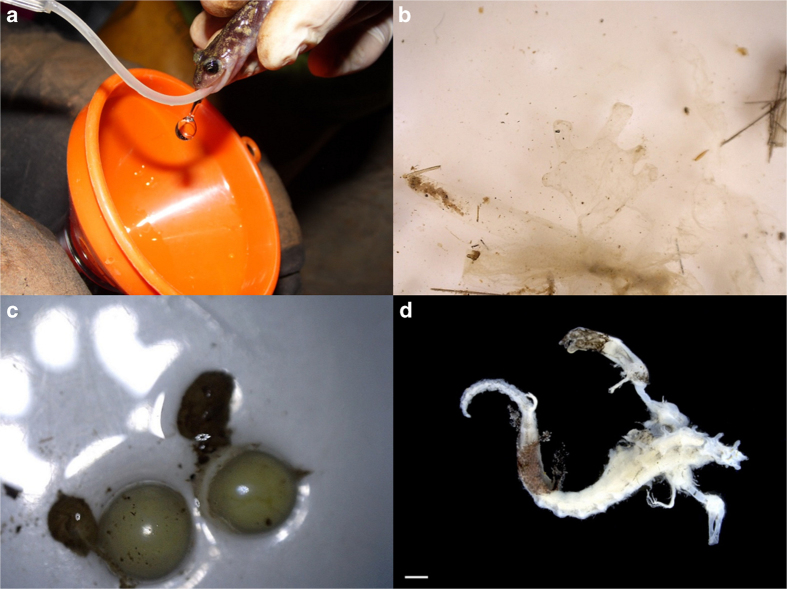
Stomach flushing and details of vertebrate prey items. (**a**) *Hydromantes* underwent stomach flushing; (**b**) *Hydromantes*’ skin found in the stomach contents (detail); (**c**) two *Hydromantes* eggs found in the stomach of a female of *H. imperialis*; (**d**) dorsal view of a juvenile *Hydromantes* eaten by a *H. ambrosii* female (bar=1 mm).

We used stomach flushing to obtain salamander stomach contents; a harmless technique already tested on *Hydromantes* salamanders^34^. We used a 5 ml syringe filled with tap water; where the needle was replaced by a plastic pipe of 1 mm diameter. Salamander was first positioned upside down (Fig. 2a) and the free extremity of the pipe was carefully inserted into its mouth, reaching the stomach. Once the pipe was in position, water was gently injected while salamander’s belly was massaged; the reflux was collected in a collection tube using a small funnel. Stomach flushing was performed only on salamanders with SVL≥30 mm, as this method could be too invasive for small individuals. In larger salamanders (SVL≥70 mm) when the first reflux was without content, the flushing was repeated one more time in order to confirm the stomach emptiness. The stomach content was fixed in 75% ethanol. After flushing, salamanders were released at the point of captured.

### Stomach contents analysis

**Figure 3:**
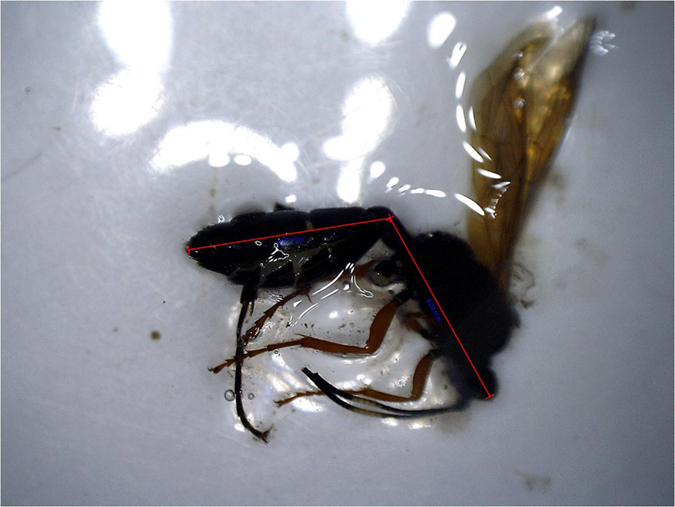
Example of prey item measurement.

Stomach contents were examined in the lab using an optical microscope. Prey items were recognised (when possible) at the order level, with the exception of the Staphylinidae (among Coleoptera) and Formicidae (among Hymenoptera) which were considered separately because of their peculiar ecology along with easy morphological identification; the general works of Sabelli and Chinery^35,36^ were taken as guiding references for the identification of the different consumed prey items. In some cases, it was also possible to distinguish arthropods' at their different stages of development (for more information see Data Records). We defined three different categories of "stomach content": "empty", without prey; "not-identifiable", when the advanced stage of digestion prevented any identification to at least the order level; "full", when at least one prey item was recognizable. For each full stomach content the minimum number of recognizable items was counted according to (ref. 5). For each typology of prey item, we counted the prey items as the sum of *a*) whole prey, *b*) heads, *c*) residual of single abdomens and single heads (only when abdomens > heads). Considering that stomach contents often contained numerous prey segments, when single appendices were recognisable with confidence (e.g., pincers, elytra), and no head/abdomen were matching with them, we added to the previous count the half (rounded up) of the appendices sum. Using a digital microscope (MAOZUA 5MP 20×–300×) we took pictures of the intact prey items and measured the maximum length (mm) using a built-in software (Fig. 3).

### Code availability

No code was used in this study.

## Data Records

The first dataset (Data on the diet of *Hydromantes* salamanders, Data Citation 1) consists of:

1. 1,250 salamander samples from 6 different *Hydromantes* species (average 208.33±10.35 individuals per species), divided in a) 319 individuals with empty stomach (average±SD per species; 53.17±38.93), b) 370 with non-recognizable contents (61.67±39.93) and c) 561 individuals with full stomach (93.5±48.74). Given that each population was sampled up to 4 times, and individuals were not marked, each individual could be present more than ones in the dataset.
2. 5,996 recognized invertebrate prey items (average±ES per individual, 10.69±0.70) belonging to 40 different taxa (Pulmonata, Sarcoptiformes, Mesostigmata, Trombidiformes, Araneae, Pseudoscorpiones, Opiliones, Lithobiomorpha, Geophilomorpha, Scolopendromorpha, Julida, Polydesmida, Isopoda, Symphypleona, Poduromorpha, Entomobryomorpha, Zygentoma, Ephemeroptera, Odonata_ninfa, Orthoptera, Blattodea, Psocoptera, Hemiptera, Endopterygota_larva, Hymenoptera, Hymenoptera_Formicidae, Coleoptera, Coleoptera_Staphylinidae, Coleoptera_larva, Neuroptera, Trichoptera_larva, Lepidoptera, Lepidoptera_larva, Diptera, Diptera_larva, Archaeognatha, Tricladida, Gordea, Nematoda, Haplotaxida).
3. 10 recognized Vertebrate's prey items: 6 skin residuals, 3 eggs and 1 *Hydromantes* juvenile (Fig. 2b–d).
4. NA means no specific data existing. SVL and position were not always recorded; in case of empty stomachs, NA was added to all other columns; if contents were not identifiable, NA was added to all prey typologies.

Detailed explanation of dataset "Data on the diet of *Hydromantes* salamanders" (Data Citation 1) is given in Table 1.

The second dataset ("Measures of intact prey items", Data Citation 1) consists of:

1. 352 intact invertebrate prey items measured.

Detailed explanation of dataset "Measures of intact prey items" (Data Citation 1) is given in Table 2.

## Technical Validation

Sites were surveyed once per season to avoid individual resampling. During each season, all surveys were performed within 30 days to limit variation of climate conditions, which can in turn affect prey composition. Considering differences in environmental conditions characterizing the studied sites, populations could show different phenology; indeed, during our surveys, within the same period in some cases we observed high population densities while in others no individuals were observed. Surveys on multiple years and season were performed to avoid biased data collection^37^. Blinded stomach contents analyses were performed to further reduce any possible bias^38^.

## Usage Notes

Dataset is provided in CSV format, which can be used with the free statistic program R^39^. Before starting the analyses, the linear distance of individuals from the cave entrance should be square-root transformed (hereafter, depth), while the number of prey items should be log transformed to improve normality and reduce skewness. Precise coordinates of the studied caves are not shown as species are strictly protected.

## Additional information

**How to cite this article:** Lunghi E. *et al.* Field-recorded data on the diet of six species of European *Hydromantes* cave salamanders. *Sci. Data* 5:180082 doi: 10.1038/sdata.2018.83 (2018).

**Publisher’s note:** Springer Nature remains neutral with regard to jurisdictional claims in published maps and institutional affiliations.

## Supplementary Material



## Figures and Tables

**Table 1:** Data on the diet of *Hydromantes* salamanders.

**Column**	**Data description**	**Typology of data**
1	Year	The year in which the survey was performed
2	Season	The season in which the survey was performed
3	Species	The sampled species
4	Site	The sampled site
5	Longitude	Coordinate x
6	Latitude	Coordinate y
7	Group	Salamander’s life history group (m/f/j)
8	Depth	Salamander’s linear distance from connection with surface
9	SVL	Salamander’s snout-vent length (mm)
10	Condition	Indicate if stomach was empty (1) or not (0)
11	Not_identifiable	Indicate if stomach contents were identifiable (0) or not (1)
12 to 54	Prey typology	For each prey typology the total number of recognized items is reported
Detailed information of the first dataset related the diet of *Hydromantes* salamanders.		

**Table 2:** Detailed information of the second dataset related the size of intact prey items recognized in *Hydromantes* stomach contents.

**Column**	**Data description**	**Typology of data**
1	Year	The year in which the survey was performed
2	Season	The season in which the survey was performed
3	Species	The sampled species
4	Site	The sampled site
5	Longitude	Coordinate x
6	Latitude	Coordinate y
7	Group	(males, females, juveniles)
8	SVL	Salamander’s snout-vent length (mm)
9	Typology	Indicates the typology of the prey item
10	Size	Maximum length of the prey item (mm)
